# Impact of immunochemotherapy administration sequence on overall survival in advanced esophageal and gastric cancers: a propensity score-matched multicenter analysis

**DOI:** 10.1007/s00262-026-04382-3

**Published:** 2026-04-06

**Authors:** Junjie Hang, Lixia Wu, Li Xiao, Qiuhua Duan, Han Zhao, Junjie Huang, Jinfeng Guo, Yiqing Huang, Dapeng Zhang, Gang Cheng, Xiao Dong, Peng Xue, Jianhua Chang

**Affiliations:** 1 Department of Medical Oncology, National Cancer Center/National Clinical Research Center for Cancer/Cancer Hospital and Shenzhen Hospital, Chinese Academy of Medical Sciences and Peking Union Medical College, Shenzhen, 518116 China; 2 Shanghai JingAn District ZhaBei Central Hospital, Shanghai, 200070 China; 3 The Affiliated Changzhou Second People’s Hospital of Nanjing Medical University, Changzhou Medical Center, Nanjing Medical University, Changzhou, 213000 China; 4 Nanchuan Hospital Affiliated to Chongqing Medical University/The People’s Hospital of Nanchuan Chongqing, Chongqing, 408400 Nanchuan China; 5 JC School of Public Health and Primary Care, Faculty of Medicine, The Chinese University of Hong Kong, Hong Kong SAR, China; 6 Centre for Health Education and Health Promotion, Faculty of Medicine, Chinese University of Hong Kong, Hong Kong SAR, China; 7 The Affiliated Bozhou Hospital of Anhui Medical University, Bozhou, 236000 China; 8 Department of Oncology, Shanghai General Hospital, Shanghai Jiao Tong University School of Medicine, Shanghai, 200080 China; 9 Shanghai Jiahui International Hospital Cancer Center, Shanghai, 200030 China

**Keywords:** Immunochemotherapy administration sequence, Overall survival, Esophageal cancers, Gastric cancers, Propensity score-matched analysis

## Abstract

**Background:**

The optimal sequence of administering immune checkpoint inhibitors (ICIs) and chemotherapy in advanced esophageal cancer (EC) and gastric cancer (GC) remains to be established. Understanding how this sequencing affects overall survival (OS) is crucial for optimizing treatment strategies.

**Methods:**

This multicenter study enrolled 334 patients diagnosed with advanced EC or GC between January 2018 and October 2024. To reduce confounding biases, propensity score matching (PSM) was applied, resulting in 292 matched patients categorized into two groups, including those receiving ICIs first (IF) and those receiving chemotherapy first (CF).

**Results:**

Following PSM, Kaplan–Meier analysis showed that the CF group had a significantly prolonged median OS compared to the IF group (16.80 vs. 14.60 months; *p* = 0.014). These findings were further corroborated in the unmatched cohort (16.60 vs. 14.60 months; *p* = 0.012). Multivariable analysis identified ECOG performance status, PD-L1 expression, antibiotic use, and line of ICI treatment as independent prognostic factors, whereas the ***i****mmunochemotherapy administration sequence* did not retain independent prognostic significance. Subgroup analysis revealed that among patients receiving first-line immunochemotherapy, those in the CF group had significantly longer OS (16.50 vs. 12.30 months; *p* = 0.004), with no significant difference observed for those beyond first-line treatment (*p* = 0.862). The objective response rate and disease control rate were comparable between the two groups.

**Conclusions:**

This study suggests that administering chemotherapy prior to immunotherapy appears to improve OS in patients with advanced EC and GC, particularly when initiated as first-line treatment. These findings highlight the need for further research into biomarkers and personalized treatment strategies to enhance patient outcomes.

**Supplementary Information:**

The online version contains supplementary material available at 10.1007/s00262-026-04382-3.

## Introduction

The introduction of immune checkpoint inhibitors (ICIs), particularly those targeting the programmed cell death protein 1 (PD-1) and its ligand PD-L1, has revolutionized cancer treatment by leveraging the host immune system to combat malignancies [1]. The PD-1/PD-L1 axis suppresses T cell activation by transmitting inhibitory signals upon binding, whereby blocking this interaction can restore antitumor immunity and prevent T cell exhaustion [2]. For gastric cancer (GC) and esophageal cancer (EC), emerging evidence indicates that immunotherapy has become an integral component of standard treatment protocols. The Checkmate-649 trial demonstrated the efficacy of PD-1 inhibitors as a first-line treatment for advanced GC and EC, through the combined use of nivolumab and chemotherapy [3]. The median over survival (OS) for the nivolumab plus chemotherapy group was 13.1 months, which was significantly longer than the 11.1 months observed with chemotherapy alone. Subsequently, the KEYNOTE-811 trial investigated the effectiveness of first-line pembrolizumab in combination with trastuzumab and chemotherapy for HER2-positive advanced GC [4]. The KEYNOTE-590 trial further confirmed the superiority of pembrolizumab combined with chemotherapy as a first-line treatment for advanced EC [5].

Despite these success, clinical responses to ICIs remain heterogeneous. Several studies have demonstrated that, for a subset of patients, ICIs can induce durable responses, generating long-lasting specific immunological memory against tumors [6, 7]. The response to ICIs is influenced by a variety of factors, including the histopathological features of tumor [8–10], the molecular profile of the cancer [11–14], and clinical characteristics of patients. These characteristics encompass the Eastern Cooperative Oncology Group Performance Status (ECOG PS) [15–17], the sites of metastases [18, 19], previous treatments [20, 21], and external interventions, such as potential drug interactions affecting the immune system [22].

Emerging evidence suggests that several major cytotoxic drugs, such as carboplatin, cisplatin, fluorouracil, irinotecan, oxaliplatin, and paclitaxel, can upregulate PD-L1 expression on cancer cells. This modulation occurs through the generation of danger signals and promotes antitumor immunogenicity by activating cytotoxic T lymphocytes, facilitating the maturation of antigen-presenting cells, depleting immunosuppressive regulatory T cells, and expanding myeloid-derived suppressor cells [23]. The combination of immunocompatible cytotoxic drugs combined with anti-PD1 antibodies represents therapeutic strategy aimed at enhancing direct tumor cell killing while minimizing the activation of immunosuppressive pathways and cancer cell survival mechanisms. Most cytotoxic agents are classified as inducers of immunogenic cell death (ICD) and possess immune-regulatory functions through these mechanisms [24]. However, the optimal sequence for combining ICIs with chemotherapy has not been deeply explored and it remains a topic of active debate. A preclinical study has demonstrated that the delivery of PD-1 2 days after chemotherapy showed the strongest synergistic effect of immunotherapy in various tumor models, including malignant melanoma model, lung metastasis model, and intraperitoneal metastasis model [25]. It was also found that administering the anti-PD-1 Ab 3 days after chemotherapy resulted in the least damage to lymphocytes in vivo from another study [26]. But a preclinical study showed that, compared with other treatments, administering anti-PD-L1 followed by oxaliplatin can increase the CD8 + T cell population and the ratio of T effector cells to Treg, thus significantly increased survival in vivo and inhibited tumor growth in tumor-bearing mice [27]. Therefore, it is necessary to seriously consider the difference in clinical efficacy brought by the sequential administration of immunochemotherapy to patients, so as to better optimize the treatment plan.

The aim of our study is to investigate how the sequence of immunochemotherapy administration affects treatment outcomes in patients with advanced EC and GC. We employed one-to-one propensity score matching (PSM) and multivariate Cox proportional hazards regression analysis to minimize bias as much as possible.

## Methods

### Patient selection

This retrospective multicenter cohort study included 363 patients diagnosed with advanced EC or GC between January 1, 2018, and October 31, 2024, across five institutions including Cancer Hospital & Shenzhen Hospital, Shanghai General Hospital, the Affiliated Changzhou Second People’s Hospital of Nanjing Medical University, the Affiliated Bozhou Hospital of Anhui Medical University, and Shanghai JingAn District ZhaBei Central Hospital. Eligibility criteria included histologically confirmed gastric adenocarcinoma or esophageal squamous cell carcinoma, complete clinical and pathological data, and follow-up information for survival outcomes. Additional inclusion criteria required evaluable lesions per Response Evaluation Criteria in Solid Tumors (RECIST), version 1.1, and an ECOG PS of 0 to 2. Exclusion criteria included a history of autoimmune diseases or active autoimmune diseases; receiving ongoing systemic immunosuppressive therapy; and other concurrent malignancies. Patients’ written informed consents and approval from the Institutional Review Boards of Cancer Hospital & Shenzhen Hospital (YW2025-65-1) were obtained for the use of these clinical materials.

### Data collection and variables

We reviewed de-identified electronic medical records to extract treatment-related variables, including the administration time of ICI, the interval between chemotherapy and ICI, the immunochemotherapy administration sequence, and other clinicopathological characteristics at the time of the first infusion of immunochemotherapy. The primary outcome of this study was OS, defined as death from any cause, measured from the date of the first infusion of immunochemotherapy. Patients lost to follow-up were censored at the time of last known contact.

### PD-L1 immunohistochemical assays

As we mentioned in the previous study, the PD-L1 assays (22C3) was performed by its corresponding autostainer (Dako Autostainer Link48 for 22C3 assays) according to the manufacturers’ instructions. The combined positive score (CPS) was calculated by dividing the number of PD-L1-stained cells (tumor cells, lymphocytes, macrophages) by the total number of viable tumor cells, multiplied by 100. The maximum combined positive score was defined as 100. The areas with tumor necrosis were exempted for the assessment.

### Statistical analysis

Statistical analyses were conducted using R software (version 3.3.1, Institute for Statistics and Mathematics, Vienna, Austria) and SPSS statistical software (version 23.0, SPSS Inc, IBM, Armonk, NY, USA). Continuous variables were summarized as medians with interquartile ranges and were compared using the Mann–Whitney U test. Categorical variables were expressed as frequencies (%) and analyzed via chi-square or Fisher’s exact tests as appropriate.

To minimize confounding bias, one-to-one PSM was conducted using logistic regression to estimate propensity scores based on clinically relevant covariates, including ECOG PS at ICI initiation and the interval between chemotherapy and ICI. A nearest-neighbor algorithm with a caliper width of 0.2 standard deviations of the logit propensity score was applied to enhance comparability of the infusion groups, thereby improving the rigor of the survival analysis, without replacement. After matching, 292 patients (146 matched pairs) were retained for analysis. Balance between groups was assessed using standardized mean differences (SMD), with an SMD of < 0.1 indicating adequate balance (Supplementary Fig. 1).

Survival outcomes were estimated using the Kaplan–Meier method and compared with log-rank tests. Univariable and multivariable Cox proportional hazards regression models were employed to identify independent prognostic factors. Variables with *p* < 0.10 in the univariable analysis were included in the multivariable model. Hazard ratios (HRs) and 95% confidence intervals (CIs) were calculated.

## Results

**Fig. 1:**
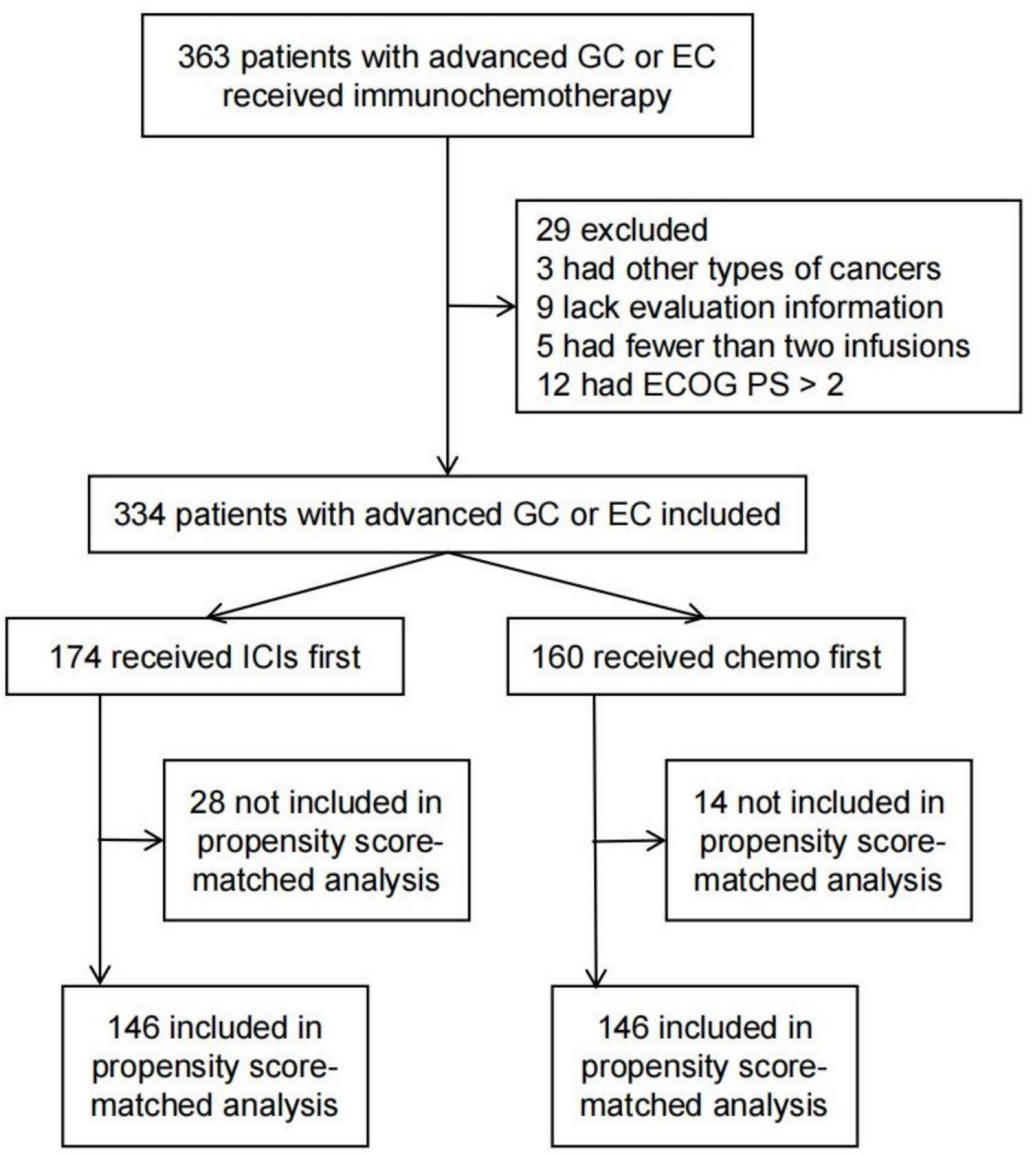
Study profile

A total of 200 patients with advanced gastric cancer and 134 patients with advanced esophageal cancer treated with immunochemotherapy were included in this study. Among them, 174 patients received ICIs first (IF) and 160 patients received chemotherapy first (CF) (Fig. 1). In the complete unmatched sample, there were 247 male patients (74.0%) and 87 female patients (26.0%), with a median age of 67 years. At the time of ICI initiation, a lower proportion of patients in the IF Group had an ECOG PS of 0 or 1 (58.0%, *n* = 101) compared to the CF Group (68.1%, *n* = 109, *p* = 0.057). Furthermore, the percentage of patients receiving immunochemotherapy on the same day was significantly higher in the IF Group (56.9%) than in the CF Group (45.0%, *p* = 0.030). No other significant imbalances in characteristics between the two groups were observed (Supplementary Table 1).

**Fig. 2:**
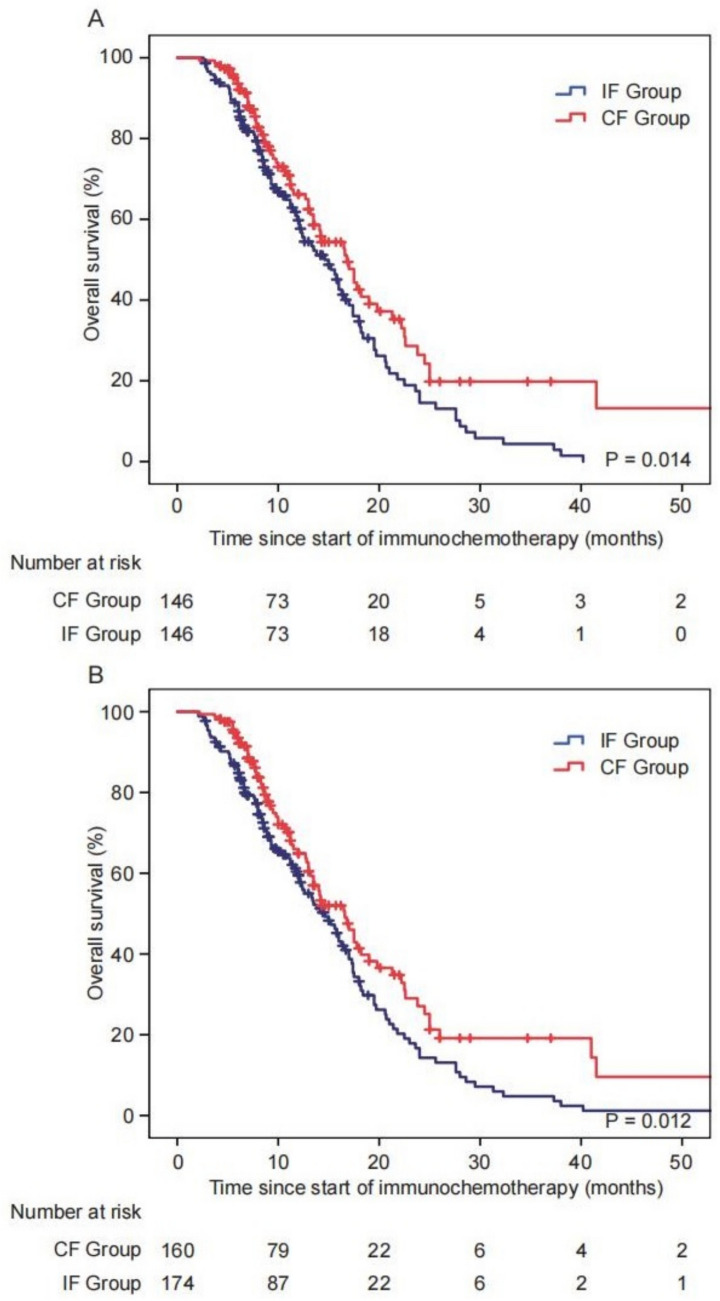
Overall survival for propensity score-matched groups (**a**) and unmatched groups (**b**)

After PSM based on ECOG PS at ICI initiation and the interval between chemotherapy and ICI, 146 patients (50%) from each group remained, comprising 73 women (25%) and 219 men (75%), with a median age of 67 years. Clinical and pathological characteristics were well balanced between the two groups (*p* > 0.05, Table 1). In the propensity score-matched populations, patients in the CF Group exhibited longer OS than those in the IF Group, with a median OS of 16.80 months (95% CI 13.72–19.88) versus 14.60 months (95% CI 11.98–17.22); *p* = 0.014, Fig. 2a). In the unmatched group, survival analysis further demonstrated that patients in the CF Group had significantly prolonged OS compared to the IF Group, with a median OS of 16.60 months (95% CI 13.85–19.35) versus 14.60 months (95% CI 12.18–17.02; *p* = 0.012, Fig. 2b).

**Table 1:** Baseline patient characteristics in the propensity score-matched population

Characteristics	ICI first (*n* = 146)	Chemotherapy first (*n* = 146)	*p* value
*Demographic characteristics*			
Age at ICI initiation (years)	67 (27–89)	67 (25–85)	0.910
Sex			
Male	116 (79.5%)	103 (70.5%)	0.079
Female	30 (20.5%)	43 (29.5%)	
ECOG PS at ICI initiation			
0–1	94 (64.4%)	95 (65.1%)	0.903
2	52 (35.6%)	51 (34.9%)	
DM			
Yes	13 (8.9%)	12 (8.2%)	0.834
No	133 (91.1%)	134 (91.8%)	
*Tumor variables*			
Tumor type			
Esophageal cancer	56 (38.4%)	69 (47.3%)	0.124
Gastric cancer	90 (61.6%)	77 (52.7%)	
Distant metastasis			
Yes	111 (76.0%)	103 (70.5%)	0.290
No	35 (24.0%)	43 (29.5%)	
PD-L1 expression (CPS)			
≥ 1%	49 (33.6%)	50 (34.2%)	0.705
< 1%	46 (31.5%)	42 (28.8%)	
Missing	51 (34.9%)	54 (37.0%)	
*ICI treatment variables*			
Antibiotic use			
Yes	19 (13.0%)	18 (12.3%)	0.860
No	127 (87.0%)	128 (87.7%)	
Corticosteroids use			
Yes	65 (44.5%)	54 (37.0%)	0.190
No	81 (55.5%)	92 (63.0%)	
Pain management			
Yes	53 (36.3%)	49 (33.6%)	0.623
No	93 (63.7%)	97 (66.4%)	
Use of NSAIDs			
Yes	25 (17.1%)	23 (15.8%)	0.752
No	121 (82.9%)	123 (84.2%)	
Use of strong opioids			
Yes	26 (17.8%)	28 (19.2%)	0.763
No	120 (82.2%)	118 (808%)	
Chemotherapy regimens			
Fluorouracil	26 (17.8%)	21 (14.4%)	0.430
Paclitaxel	24 (16.4%)	20 (13.7%)	
Fluorouracil + platinum	48 (32.9%)	49 (33.6%)	
Paclitaxel + platinum	48 (32.9%)	56 (38.4%)	
ICI regimens			
Carilizumab	57 (39.0%)	56 (38.4%)	0.619
Sintilimab	49 (33.6%)	53 (36.3%)	
Tislelizumab	33 (22.6%)	34 (23.3%)	
Nivolumab/pembrolizumab	7 (4.8%)	3 (2.1%)	
ICI treatment line			
Firstline	110 (75.3%)	110 (75.3%)	0.948
Secondline	31 (21.2%)	30 (20.5%)	
3rd, or 4th-line	5 (3.4%)	6 (4.1%)	
Administration time of ICI			
Morning	112 (76.7%)	103 (70.5%)	0.232
Afternoon	34 (23.3%)	43 (29.5%)	
Interval between chemotherapy and ICI			
On the same day	71 (48.6%)	72 (49.3%)	0.907
At least one day apart	75 (51.4%)	74 (50.7%)	
*Laboratory variables*			
ALC (*10^9/L)	1.17 (0.37–2.66)	1.18 (0.19–18.20)	0.099
AMC (*10^9/L)	0.46 (015–1.89)	0.45 (0.01–1.92)	0.442
ANC (*10^9/L)	3.54 (0.80–15.83)	3.63 (1.02–15.50)	0.059
HGB (g/L)	113 (63–167)	115.5 (62–166)	0.223
CEA (ng/mL)	3.09 (0.26–1000.00)	2.74 (0.22–1000.00)	0.890

*ICI* immune checkpoint inhibitor, *ECOG* Eastern Cooperative Oncology Group, *PS* Performance Status, *CPS* Combined Positive Score, *NSAIDs* non-steroidal anti-inflammatory drugs; *5-Fu* 5-Fluorouracil, *Pac* Paclitaxel, *ALC* absolute lymphocyte count, *AMC* absolute monocyte count, *ANC* absolute neutrophil count, *HGB* hemoglobin, *CEA* carcinoembryonic antigen

Univariate Cox regression analysis revealed that several factors, including ECOG PS at ICI initiation, tumor type, PD-L1 expression, antibiotic use, corticosteroids use, ICI treatment line, immunochemotherapy administration sequence, and CEA levels, were significantly associated with OS (*p* < 0.10, Table 2). These variables were further included in the multivariate analysis, which identified ECOG PS at ICI initiation, PD-L1 expression, antibiotic use, and ICI treatment line as independent prognostic factors (*p* < 0.05). However, the immunochemotherapy administration sequence did not demonstrate independent prognostic value in multivariate analysis (*p* = 0.054).

**Table 2:** Clinicopathological characteristics associated with overall survival

	Univariate analysis			Multivariate analysis		
Characteristics	OR	95%CI	*p* value	OR	95%CI	*p* value
Age						
< 65	1.056	0.775–1.440	0.730			
> 65	Reference					
Sex						
Male	1.140	0.801–1.623	0.467			
Female	Reference					
ECOG PS at ICI initiation						
2	1.951	1.411–2.697	< 0.001	1.819	1.198–2.762	0.005
0–1	Reference					
DM						
Yes	0.920	0.497–1.703	0.791			
No	Reference					
Tumor type						
Gastric cancer	1.393	1.016–1.908	0.039	1.289	0.848–1.959	0.235
Esophageal cancer	Reference					
Distant metastases						
Yes	1.176	0.825–1.676	0.371			
No	Reference					
PD-L1 expression						
>1%	0.478	0.324–0.705	< 0.001	0.573	0.377–0.869	0.009
< 1%	Reference					
Antibiotic use						
Yes	1.794	1.141–2.822	0.011	3.364	1.893–5.981	< 0.001
No	Reference					
Corticosteroids use						
Yes	1.308	0.959–1.783	0.090	1.186	0.775–1.814	0.433
No	Reference					
ICI treatment line						
1st-line	0.718	0.511–1.008	0.056	0.487	0.304–0.782	0.003
>2nd-line	Reference					
Administration time of ICI						
Morning	0.130	0.718–0.936	0.718			
Afternoon	Reference					
Interval between chemotherapy and ICI						
On the same day	0.777	0.570–1.059	0.110			
At least one day apart	Reference					
Immunochemotherapy administration sequence						
Chemotherapy first	0.677	0.494–0.927	0.015	0.667	0.441–1.007	0.054
ICI first	Reference					
HGB (g/L)	0.999	0.992–1.007	0.845			
ANC (*10^9/L)	1.005	0.950–1.062	0.871			
ALC (*10^9/L)	0.849	0.671–1.073	0.170			
AMC (*10^9/L)	0.832	0.412–1.679	0.608			
CEA (ng/ml)	1.002	1.000–1.003	0.014	1.001	0.998–1.003	0.621

**Fig. 3:**
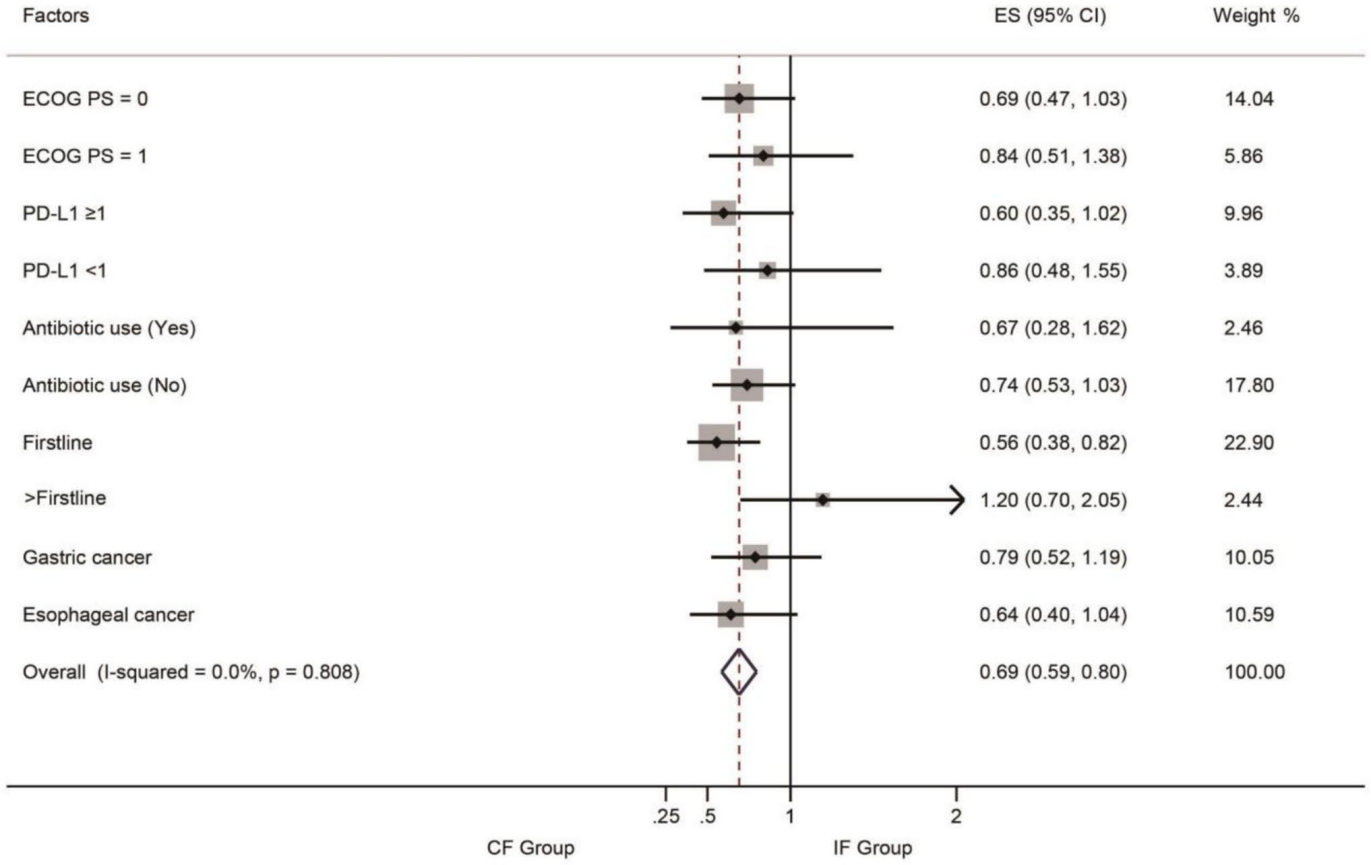
Forest plot of immunochemotherapy administration sequence hazard ratios in the subgroup analysis

Additionally, we conducted a subgroup analysis of the immunochemotherapy administration sequence (Fig. 3), which indicated that OS of the CF Group was significantly prolonged compared to the IF Group among patients receiving first-line immunochemotherapy, with a median OS of 16.50 months (95% CI 12.67–20.33) versus 12.30 months (95% CI 10.58–14.02; *p* = 0.004). However, no significant difference in OS was found between the two groups for patients receiving more than first-line immunochemotherapy (*p* = 0.862). Notably, we found that specific patients, stratified by ECOG PS = 0, PD-L1 expression ≥ 1, the absence of antibiotics use, and tumor type (EC), belonged to the CF Group were prone to have a longer OS (Fig. 3).

We also evaluated the effect of sequence of immunochemotherapy on the efficacy of regimes and found that patients in the CF Group had almost the same likelihood of response to chemoimmunotherapy compared with those in the IF Group (CR, 1.37% vs. 2.05%; PR, 34.93% vs. 34.25%; SD, 38.36% vs. 39.04%; PD, 25.34% vs. 24.66%). Overall, patients in the CF Group had almost the same objective response rate (ORR; 36.30% vs. 36.30%) and disease control rate (DCR; 74.66% vs. 75.34%) compared with those in the IF Group, without significant difference in the efficacy of chemoimmunotherapy.

## Discussion

After conducting PSM and Kaplan–Meier analysis of 292 patients with advanced EC and GC, we found that the immunochemotherapy administration sequence was associated with the OS of these patients. Subgroup analysis of the immunochemotherapy administration sequence further showed that OS of the CF Group was significantly prolonged compared to the IF Group among patients receiving first-line immunochemotherapy. However, subsequent multivariate analysis revealed that immunochemotherapy administration sequence did not have independent prognostic value.

ICIs are known to exert tumoricidal activity indirectly by reinvigorating antitumor T cells [28]. The clinical efficacy of various chemotherapies has been linked to their role in stimulating anticancer immunity, either by initiating the release of immunostimulatory molecules from dying cancer cells or by producing off-target effects on immune cell populations [24]. The combination of ICIs with chemotherapy has significantly enhanced the therapeutic efficacy of cancer treatment [29, 30]. Furthermore, combining immunotherapy with specific chemotherapeutic agents can yield greater efficacy than either treatment alone [31]. However, investigations examining the effect of different immunochemotherapy administration sequence on patients’ prognosis remain limited.

In vitro studies showed that administering the anti-PD-1 antibody three days after chemotherapy, specifically with dicycloplatin, resulted in significantly weaker cytotoxic effects on lymphocytes compared to administration of the anti-PD-1 antibody either before or concurrently with chemotherapy. Additionally, a retrospective analysis 64 lung cancer patients receiving PD-1/PD-L1 inhibitors in combination with chemotherapy as second- or higher-line therapy indicated that administering PD-1/PD-L1 inhibitors 1 to 10 days (especially 3 to 5 days) after chemotherapy is superior to administration prior to or concurrently with chemotherapy in patients with refractory lung cancer [26]. Huo et al. found that in advanced non-small cell lung cancer, PD-1 expression on CD8 + T cells was downregulated on day 1 and 2, but recovered by day 3 after chemotherapy, a process mediated by the calcium influx-P65 signaling pathway. Moreover, PD-L1 expression in myeloid-derived suppressor cells was significantly reduced on day 3. RNA sequencing analysis indicated that T cell function began to gradually recover by day 3, rather than on day 1. Furthermore, both ex vivo and in vivo studies have shown that anti-PD-1 treatment on day 3 after chemotherapy may enhance the antitumor response and significantly inhibit tumor growth [32]. However, the effects of different immunochemotherapy administration sequence on patients’ prognosis in EC and GC remain unreported.

We observed a significant survival benefit for the CF group in the Kaplan–Meier analysis and specific subgroups, which was not maintained as an independent prognostic factor in the multivariable Cox regression. This discrepancy suggests that while a raw survival difference exists between the two sequences, it may be partially mediated or confounded by other clinical variables included in the model, such as ECOG PS. In the multivariable setting, these established prognostic markers account for a large proportion of the variance in survival, which can diminish the independent statistical power of the treatment sequence. Therefore, while the sequencing effect is clinically noteworthy in specific contexts, it should not be viewed as a standalone predictor of outcome in the same way as traditional staging or ECOG PS. This further underscores that treatment sequencing is likely part of a complex interplay of factors rather than a primary driver of prognosis.

The subgroup analysis results indicate that OS in the CF Group was significantly prolonged compared to the IF Group among patients receiving first-line immunochemotherapy. However, no significant difference in OS was found between the two groups for patients receiving treatment beyond the first line. This discrepancy may be attributed to the dynamic plasticity of the TME during the initial treatment phase. Recent studies have shown that select chemotherapy regimens act as extensive modifiers of the TME, leading to the reprogramming of immune responses [33]. Specifically, ICD is a form of tumor cell death that primes an anticancer immune response [34]. Conventional chemotherapeutics have been demonstrated to function as ICD inducers, releasing tumor antigens that can modulate tumor infiltrating lymphocytes (TILs) and reactivating antitumor immunity within an immunosuppressive microenvironment [35]. Consequently, this process reshapes the TME and enhances the efficacy of subsequent PD-1/PD-L1 inhibitors. Thus, our study suggests that the immune activation effect of chemotherapy may be a key mechanism underlying these findings. In future, single-cell sequencing and dynamic biopsy techniques will be used to further analyze the evolutionary patterns of TME components throughout the treatment sequence. Additionally, we will explore the predictive value of biomarkers such as CD8+T cell clonal expansion and the dynamic expression of PD-L1.

In the post-treatment period, no significant difference in OS was observed between the IF and CF groups. Several factors may be attributed to this finding. First, after multiple lines of treatment, tumor heterogeneity significantly increases, and the loss of antigen epitopes due to immune editing may diminish the effectiveness of T cell recognition [36]. Additionally, advanced-stage patients often experience T cell exhaustion and an imbalance between T cell reactivation and tumor burden, which can increase the threshold for an immune treatment response [37]. Moreover, changes in chemotherapy dosage or regimens during the post-treatment phase, such as switching to non-immunologically synergistic drugs, may also impact the immunomodulatory effects of the treatment. Therefore, optimizing treatment strategies in the post-treatment phase to enhance therapeutic efficacy necessitates further investigation. Future research should focus on exploring new biomarkers, developing personalized treatment plans, and integrating complementary treatment modalities to improve the prognosis for patients with EC and GC.

However, our study has several limitations due to its retrospective nature. It includes associated factors such as ECOG PS at ICI initiation and the interval between chemotherapy and ICI and the type of anti-PD1/PD-L1 agent administered. Given that patients were retrospectively grouped according to the immunochemotherapy administration sequence, the influence of confounding factors is unavoidable. While PSM helps balance some covariates between the groups, it cannot account for all confounders that may still influence the outcomes. These unmeasured factors, such as differences in tumor biology, individual response to treatment, and variations in clinical management practices across the study centers, could introduce residual confounding, affecting the generalizability of our findings. Additionally, selection bias remains a concern, as patients who were selected for treatment with a specific immunochemotherapy sequence (either chemotherapy first or immunotherapy first) might have had inherent differences in factors such as disease severity, prior treatments, or physician preferences. While our use of PSM aimed to minimize these biases by matching patients on key covariates, we recognize that not all variables can be captured or perfectly matched, leaving room for potential bias.

Intriguingly, despite rigorous PSM and adjustments in multivariate regression, the immunochemotherapy administration sequence did not exhibit independent prognostic value. The loss of significance in the multivariate analysis suggests that other factors, such as tumor stage, molecular subtypes, or heterogeneity within the immune microenvironment, may play a more dominant role in the prognostic landscape. This finding underscores the complexity of treatment sequencing in gastric and esophageal cancer while highlighting the necessity to contextualize our results within the broader framework of chemoimmunotherapy research. Consequently, our findings may not be generalizable to all clinical contexts. To address these limitations, additional prospective randomized trials with well-designed protocols are warranted. Furthermore, in-depth investigations into the alterations of the immune microenvironment before and after treatment, along with mechanistic studies using murine models to elucidate the underlying immunological mechanisms, are essential for advancing our understanding.

## Conclusion

In conclusion, our findings indicate that in advanced EC and GC, patients in the CF group had a significantly longer median OS compared to those in the IF group, particularly among those receiving first-line immunochemotherapy. However, the immunochemotherapy administration sequence did not emerge as an independent prognostic factor in this patient population, and no significant differences were observed in regimen efficacy based on the sequence alone. Given the observational nature of these data, prospective randomized controlled trials are necessary to validate these observed sequencing effects and to determine their definitive role in clinical practice.

## Ethical approval

Patients’ written informed consents and approval from the Institutional Review Boards of Cancer Hospital & Shenzhen Hospital (YW2025-65-1) were obtained for the use of these clinical materials.

## Supplementary Information

Below is the link to the electronic supplementary material.

Supplementary file1 (TIFF 320 KB)

Supplementary file2 (XLS 30 KB)

## Data Availability

The datasets used and/or analyzed during the current study are available from the corresponding author on reasonable request.
