# Supplementary figures and images for: Impact of immunochemotherapy administration sequence on overall survival in advanced esophageal and gastric cancers: a propensity score-matched multicenter analysis
Source: Cancer Immunol Immunother. 2026 Apr 6;75(5):136. doi: 10.1007/s00262-026-04382-3 (PMC13053723; doi:10.1007/s00262-026-04382-3)

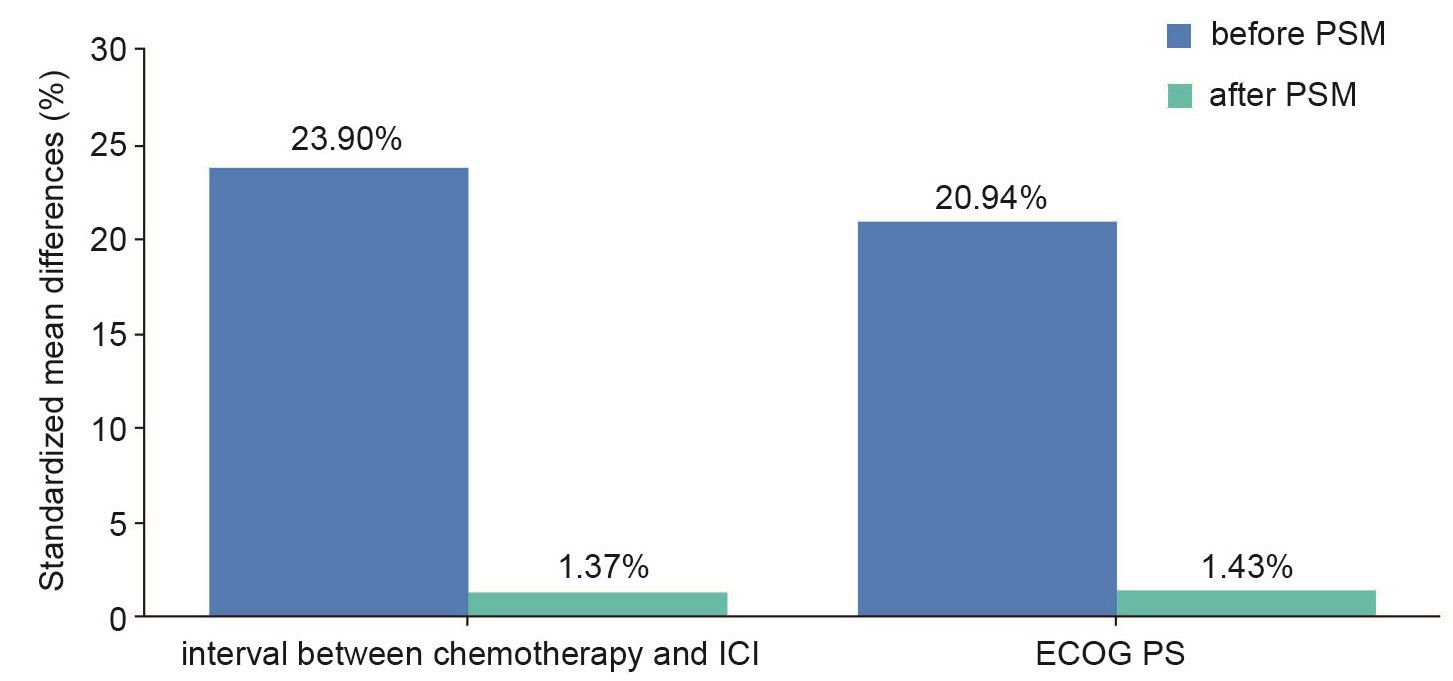

Supplement: Supplementary file 1 — Supplementary file1 (TIFF 320 KB) [file 262_2026_4382_MOESM1_ESM.tiff]
